# Identification of ᴅ-amino acid-containing peptides in human serum

**DOI:** 10.1371/journal.pone.0189972

**Published:** 2017-12-18

**Authors:** Seongmin Ha, Ingu Kim, Takumi Takata, Tadatoshi Kinouchi, Masaharu Isoyama, Minoru Suzuki, Noriko Fujii

**Affiliations:** 1 Graduate School of Science, Kyoto University, Kyoto, Japan; 2 Research Reactor Institute, Kyoto University, Osaka, Japan; 3 Protein Research Foundation, Osaka, Japan; Duke University School of Medicine, UNITED STATES

## Abstract

Biologically uncommon d-aspartate (d-Asp) residues have been shown to accumulate in proteins associated with age-related human disorders, such as cataract and Alzheimer disease. Such d-Asp-containing proteins are unlikely to be broken down completely because metabolic enzymes recognize only proteins or peptides composed exclusively of l-amino acids. Therefore, undigested d-Asp-containing peptides may exist in blood and, if detectable, may be a useful biomarker for associated diseases. In this study, we investigated d-amino acid-containing peptides in adult human serum by a qualitative d-amino acid analysis based on a diastereomer method and LC-MS/MS method. As a result, two d-Asp-containing peptides were detected in serum, both derived from the fibrinogen β-chain, a glycoprotein that helps in the formation of blood clots. One of the peptides was fibrinopeptide B, which prevents fibrinogen from forming polymers of fibrin, and the other was same peptide with C-terminal Arginine missing. To our knowledge, this is the first report of the presence of d-amino acid-containing peptides in serum and the approach described will provide a new direction on the serum proteome and fragmentome.

## Introduction

Although amino acids have two optical isomers, the l-form and the d-form, living organisms on earth are composed exclusively of l-amino acids. l-Amino acid homochirality is essential for the development and maintenance of life; therefore, historically biochemists have not paid much attention to the presence and/or function of biologically rare d-amino acids in living organisms. In recent years, however, improvements in analytical techniques have facilitated an accurate analysis of amino acid enantiomers. Consequently, d-amino acids have been detected in various living higher organisms both as a free molecule [1–6], and in peptides [7–9] and proteins [10, 11]. d-Amino acids have been conventionally detected by gas chromatography or RP-HPLC. In addition, an excellent study recently demonstrated that free d-amino acids in animal milks can be distinguished from their l-amino acid counterparts by UP-HPLC coupled with ion-mobility high-resolution MS (IM-HRMS) [12].

There are fewer reports of the detection and identification of d-amino acids in proteins and peptides because conventional methods require several steps to confirm the presence of d-Asp. (i) The protein need to be digested with a protease such as trypsin. (ii) The portion of digested proteins is separated to each peptides by RP-HPLC. (iii) To determine d/l ratios of the Asp residues, peptides are hydrolyzed with 6 M HCl and derivatization to diastereomers. (iv) The diastereoisomers are analyzed by RP-HPLC and the d/l ratio of Asp is determined by peak areas. However, this method contains a little racemization of amino acids as an artifact during hydrolysis. These problems have been recently overcome in an LC-MS analysis in which d-amino acid-containing peptides with the same mass are separated into multiple peaks on LC-MS. Because this approach does not contain the hydrolysis step, the analysis is quicker and no racemization is introduced. However, the LC-MS method also has some problems: first, not all peptides can be detected for different reasons (e.g., not ionized, mass outside detection limit); second, some isomeric peptides are not separated into multiple peaks on LC. Therefore, to accurately detect d-amino acid-containing peptides, analysis of the isomers should be performed by both methods.

Notably, d-amino acids have been widely detected in aged proteins associated with aggregation-related diseases, such as cataract [13], Alzheimer disease [14], and photo-aging of skin [15]. Among the amino acid residues in proteins, aspartate (Asp) is isomerized most frequently owing to its specific isomerization mechanism in polypeptide. Asp residues are easily inverted from the l-form to the d-form as compared with other amino acids because Asp-specific stereoinversion occurs readily via a succinimidy intermediate [16]. Indeed, d-Asp-containing crystallins have been detected in cataract samples. Because proteins containing d-amino acids have been detected in tissue samples from age-related disease, it is possible that d-amino acid-containing peptides digested from these proteins may be present in blood, representing a useful biomarker for detecting age-related disease.

Blood contains proteins secreted by various cells, glands and tissues [17]. In serum, 22 proteins including albumin, immunoglobulins, transferrin and haptoglobin make up 99% of total protein abundance [18]. In addition to these major constituents, up to 10,000 proteins that are synthesized and secreted from cells and tissues, or present as truncated fragments, are commonly found in serum, mostly at very low relative abundance [18]. The protein components of serum differ slightly depending on age, gender and genetic factors. Accordingly, several serum proteins have been identified as potential biomarkers of cardiovascular diseases, autoimmune diseases, infectious diseases and neurological disorders, among others [19–22]. Furthermore, serum contains peptide fragments from damaged tissues, which can potentially be used for the rapid and convenient diagnosis of disease by a blood test using an antibody.

It is therefore necessary for a preferably rapid and quantitative methodology that can detect low-concentration peptides as biomarkers in serum. As mentioned above, if the d-amino acid-containing peptides can be detected in human serum, they will be useful biomarkers of age-related disease. However, not only will the amount of such peptides in blood be very low, but the chemical and physical properties of d-amino acids are essentially identical to those of l-amino acids, making it difficult to specifically identify d-amino acid-containing peptide.

In this study, we have measured the amount of d-amino acids in serum peptides by a chemical method and identified the respective peptides by LC-MS/MS. To our knowledge, this is the first report of the identification of d-amino acid-containing peptides in serum. The results reported here might provide new insight into the serum proteome and fragmentome.

## Materials and methods

### Sample preparation

The study was approved by the Ethics Committee of Graduate School and Faculty of Medicine Kyoto University (R0082). Written informed consent was acquired from all donors. In total, three 4-mL blood samples from healthy donors aged ~20, ~40, and ~60 years were used in this study. Each blood sample was collected from the healthy individual in a fasting state into a plastic tube, and serum was obtained by centrifugation for 10 min at 3000*g*. To reduce the high content of protein in serum, 4.7 mL of ethanol was mixed with 2 mL of serum. After centrifugation, an aliquot of the supernatant was removed and freeze-dried. Two milliliters of 0.1% (v/v) trifluoroacetic acid (TFA) solution was then added to the remaining serum, which was applied to reversed-phase liquid chromatography (RP-HPLC).

### Isolation of peptides from human serum

Serum peptides were separated by RP-HPLC comprising a UV-970, LG-980-2, PU-980, and DG-980-50 (JASCO, Tokyo, Japan) and a C18 column (Capcell pak C18 UG 80, 3.0 x 250 mm; Shiseido, Tokyo, Japan). A linear gradient of 0%–90% acetonitrile in the presence of 0.1% TFA was applied at a flow rate of 0.5 mL/min with monitoring at 215 nm. The peptides were collected by a FRC10 fraction collector (Shimadzu, Kyoto, Japan).

### Determination of the d/l ratio of Asp in human serum-derived peptides

Aliquots (25 μL) of the RP-HPLC fractions were dried by a vacuum pump, and then hydrolyzed with 6 M gas-phase HCl at 108°C for 7 h (PicoTag Workstation; Waters, Tokyo, Japan). The hydrolyzed samples were dissolved in 0.1 M borate buffer (pH 10.4), and incubated with *o*-phthalaldehyde (OPA) and *n* (tert-butyloxycarbonyl)-l-cysteine (Boc-l-Cys) to form diastereoisomers, which were then separated by RP-HPLC using a C18 column (Nova-Pak ODS, 3.9 × 300 mm; Waters, Japan). A linear gradient of 5%–47% acetonitrile plus 3% tetrahydrofuran in 0.1 M acetate buffer (pH 6.0) was applied over 60 min at a flow rate of 0.8 mL/min at 30°C, with fluorescence detection (344 nm excitation wavelength and 433 nm emission wavelength, Shimadzu, Japan). The d/l ratios of Asp were determined by the ratio of the peak areas of the diastereoisomers.

### Peptide synthesis

Fibrinopeptide B (^1^QGVNDNEEGFFSAR^14^) was synthesized by 9-fluorenylmethyloxycarbonyl group (Fmoc)-based solid-phase peptide synthesis using an automated solid-phase peptide synthesizer (PSSM-8; Shimadzu, Japan). The coupling reaction was carried out by mixing each Fmoc amino acid (10 eq), (benzotriazol-1-yloxy)-tripyrrolidinophosphonium hexafluorophosphate (10 eq), 1-hydroxybenzotriazole hydrate (10 eq), and N-methylmorpholine (7.5 eq) in N,N-dimethylformamide (DMF). The N-terminal Fmoc group was deblocked with 30% piperidine in DMF. Spontaneous cleavage of the peptide from the resin and removal of the protective groups were achieved by treatment with a mixture containing 82.5% TFA, 5% water, 5% thioanisole, 3% ethylmethylsulfide, 2.5% 1,2-ethanedithiol, and 2% thiophenol for 6 h. The crude peptides were purified by reversed-phase high-performance liquid chromatography using a C18 column (Capcell Pak C18 ACR, 10 × 250 mm2; Shiseido, Japan) with a linear gradient of 0%−50% acetonitrile in the presence of 0.1% TFA at a flow rate of 3.0 mL/min and detection at 230 nm.

### LC-MS analysis and identification of peptides

A nanoflow HPLC system was used for LC (Paradigm MS4, Michrom BioResources, U.S.A.). MS was performed on an Ion Trap (IT) system (LCQ Fleet, Thermo, U.S.A.). All peptide samples were filtered through a 0.45-μm filter (Merck Millipore, U.S.A.) before analysis by LC-MS. Peptides were separated by a C18 column (l-column, 0.1 × 150 mm; CERI, Japan) with a linear gradient of 5%−45% acetonitrile in the presence of 0.1% formic acid applied over 45 min at a flow rate of 0.5 μL/min. All peptides were identified by Proteome Discoverer 1.0 software. MS analysis was carried out by alternating between full MS and MS/MS scan.

## Results

### Isolation of peptides from human serum

**Fig 1:**
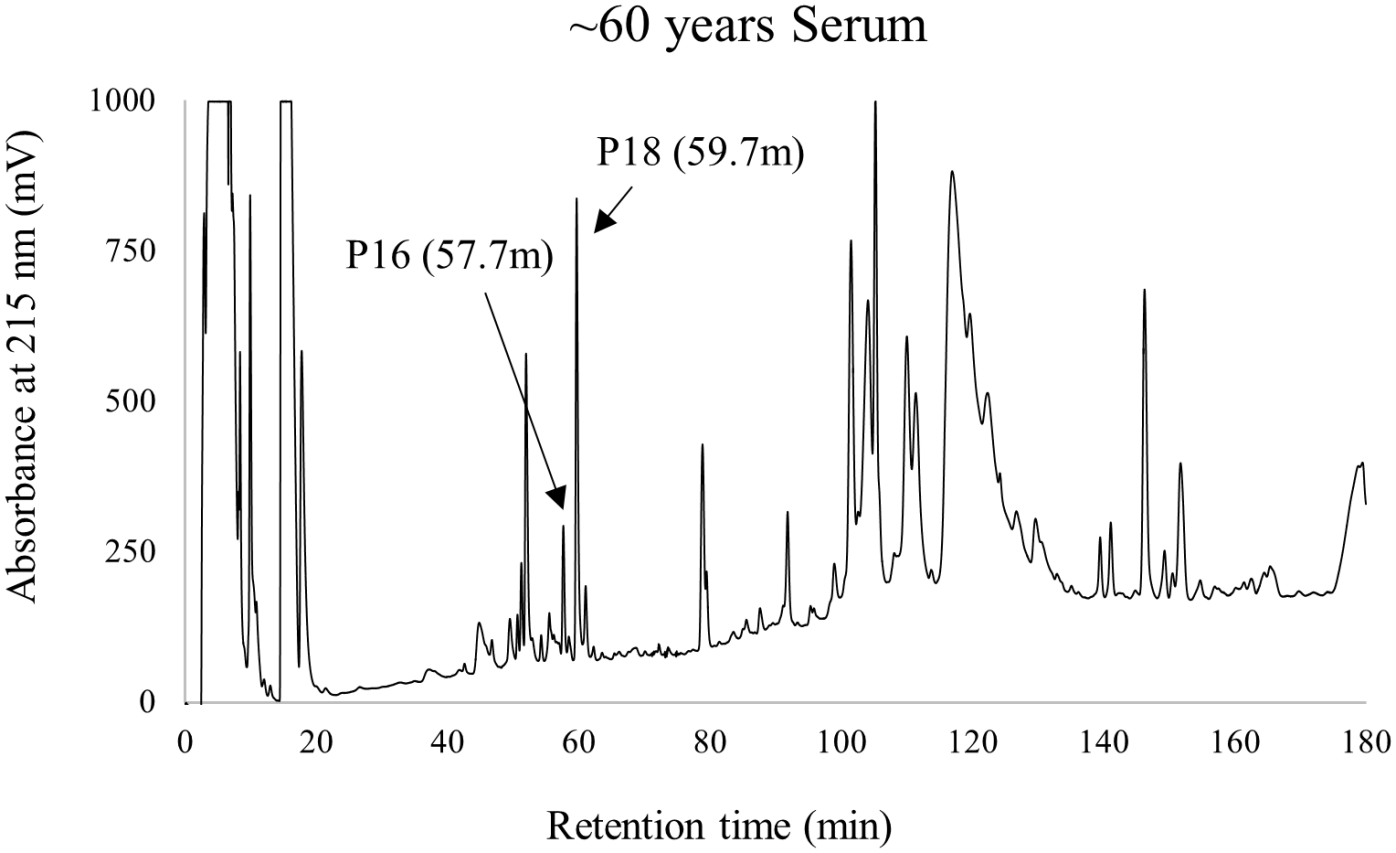
Isolation of peptides from human serum. RP-HPLC chromatogram of the serum peptides from the donor aged ~60 years. The detailed RP-HPLC conditions are described in Materials and Methods. Arrows indicate d-Asp-containing peptides.

To isolate the relatively small amount of peptides in serum, major serum proteins were first removed by using ethanol precipitation. The extracted peptides were then separated by using reversed-phase liquid chromatography (RP-HPLC). Fig 1 shows a typical RP-HPLC chromatogram of the serum peptides isolated from the donor aged ~60 years. The peptides isolated from the serum of the other aged donors showed similar chromatograms (S1 Fig). The peaks contained peptides, amino acid derivatives, lipids, and remaining serum proteins. In total, 44 peak fractions were collected across the chromatograms of serum samples from all donors.

### Determination of the d/l ratio of Asp in human serum-derived peptides

**Fig 2:**
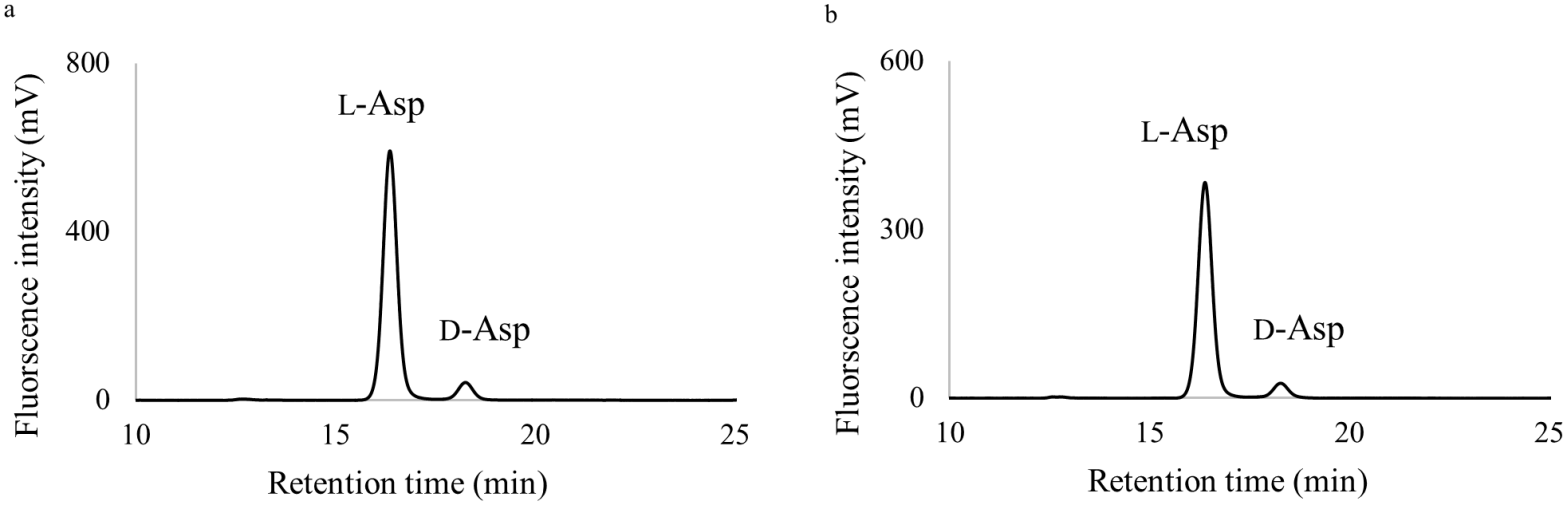
Determination of the d/l ratio of Asp in human serum-derived peptides. Elution profile of the enantiomeric separation of Asp derivatives using Boc-l-Cys-OPA. Aspartate residues from the hydrolysates of peptide peaks 16 (**a**) and 18 (**b**) in Fig 1 (serum sample from the donor aged ~60 years) are shown. The d/l ratio of Asp was estimated as 0.08 in both peaks by calculating the peak areas of the chromatograms.

To determine the extent of racemization of each peptide and its ratio, the 44 peak fractions described above were hydrolyzed to amino acids, and the resulting amino acids were derivatized to Boc-l-Cys diastereoisomers as described in Materials and Methods and analyzed by RP-HPLC. The d/l ratios of amino acids were calculated from the ratios of the peak areas of the diastereoisomers eluting in the RP-HPLC. Among 44 peptide peaks observed for the donor aged ~60 years in Fig 1, only peaks 16 and 18 contained d-Asp residues, and the d/l ratio of Asp residues in both peaks was 0.08 (Fig 2). In the other peaks, the d/l ratio of Asp residues was 0.01 to 0.02, but this was due to racemization during the hydrolysis step. To confirm how much l-Asp is converted into d-Asp during the hydrolysis step, we performed a control experiment on synthetic peptide (S2 Fig). During the hydrolysis, a maximum of 2% of l-Asp was converted to the d-form (d/l ratio, 0.02). This indicates that the actual d/l ratio of Asp residues in peaks 16 and 18 is 0.06. Two d-Asp-containing peptides were similarly detected in the serum samples from the donors aged ~ 20 and ~40 years (S3 Fig). The d/l ratio of each peptide was 0.07 to 0.08, which was similar to that of the peptides detected in serum from the donor aged ~60 years. No other d-amino acids were detected in any of the peptide fractions.

### Identification of d-Asp-containing peptides in human serum by LC-MS/MS

**Fig 3:**
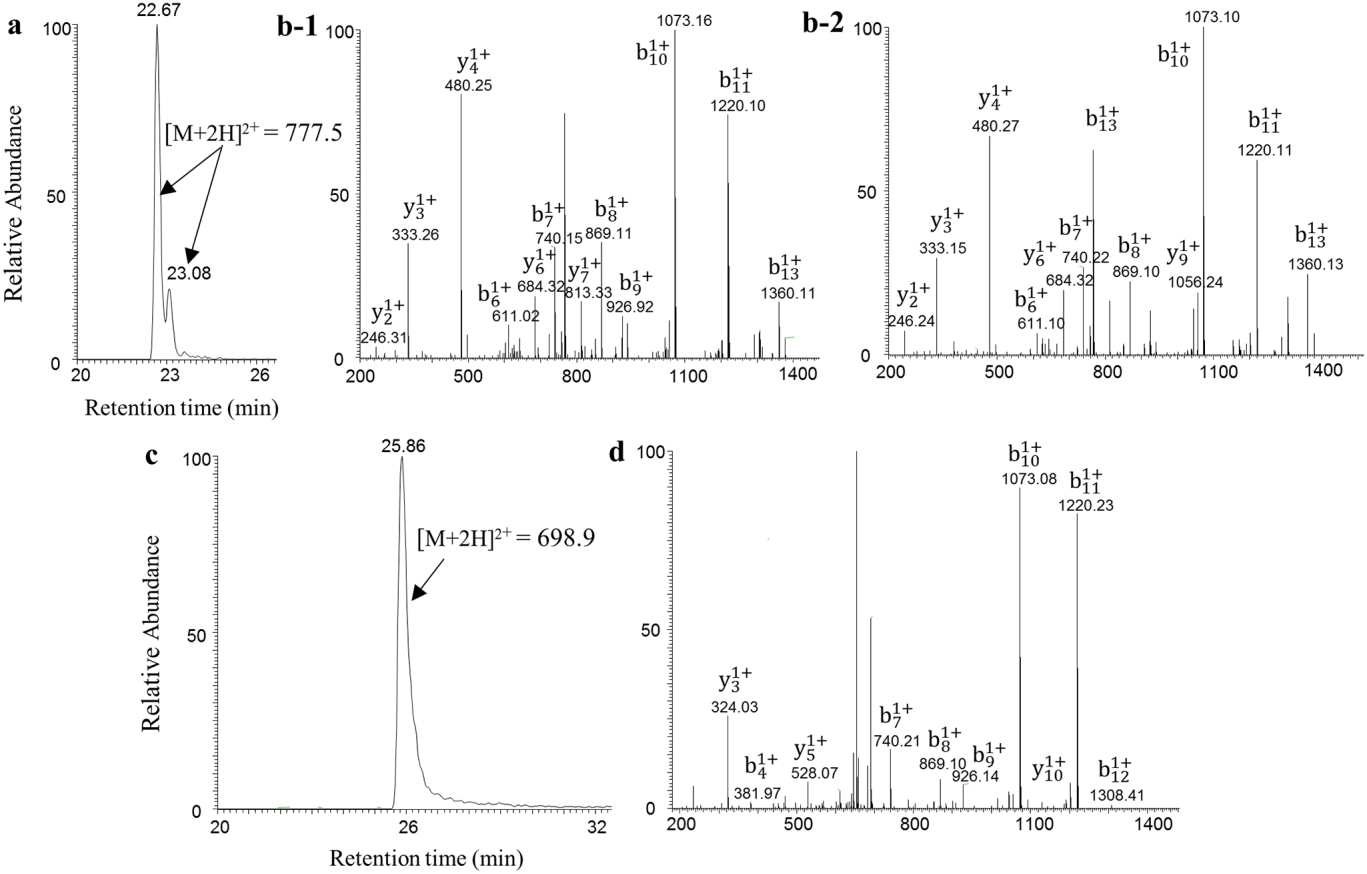
Identification of d-Asp-containing peptides by LC-MS/MS analysis. (**a**) LC-MS chromatogram of peak 16 in Fig 1. This peptide was separated into two peaks with the same mass ([M+2H]^2+^ = 777.5), indicating two isomers of the same peptide. (**b-1**) Tandem mass spectrum of the large peak in (**a**). (**b-2**) Tandem mass spectrum of the small peak in (**a**). (**c**) LC-MS chromatogram of peak 18 ([M+2H]^2+^ = 698.9) in Fig 1. (**d**) Tandem mass spectrum of the peak in (**c**). Both peptides were identified as fibrinogen β-chain-specific peptides (peak 16, ^1^QGVNDNEEGFFSAR^14^; and peak 18, ^1^QGVNDNEEGFFSA^13^). The N-terminal Gln residue was converted to pyro-Glu in both peptides.

To confirm the presence of d-Asp and identify the d-amino acid-containing peptides, LC-MS analysis was carried out. Fig 3 shows the base-peak ion chromatograms obtained from LC-MS/MS analysis of peaks 16 and 18. Peak 16 was separated into two peaks eluting at different times, 22.67 and 23.06 min, on the LC-MS chromatogram. These peaks had same MS ([M + 2H]^2+^ = 777.5) and same MS/MS product ions. As described in a previous study, d-Asp-containing peptides are separated into multiple peaks on a LC-MS chromatogram [23]. Therefore, the peak 16 peptide contains Asp isomers. Although peak 18 would also be expected to separate into two peaks owing to the presence of d-Asp, it eluted as a single peak under this condition. Peak 18 had m/z 698.9, corresponding to a doubly charged ion ([M + 2H]^2+^ = 698.9). It was confirmed that the d-Asp-containing peptides found in the serum samples from donors in their 20s and 40s had the same masses (S4 Fig). Together with the results from the diastereomeric analysis, peaks 16 and 18 were both clearly shown to contain d-Asp residues.

For each peak, the identification of peptides was performed by a combination of LC-MS and SEQUEST analyses. Peak 16 was identified as fibrinopeptide B (^1^QGVNDNEEGFFSAR^14^) and peak 18 was the sample peptide but with the C-terminal Arg14 missing (^1^QGVNDNEEGFFSA^13^). Fig 3 shows the MS/MS chromatogram of each peptide. For both peptides, N-terminal Gln residues were observed as pyro-Glu residues on the basis of –17 mass of the theoretical mass of the peptides.

## Discussion

In this study, we screened 44 peptide fractions from human serum for d-amino acid-containing peptides, and identified two peptides corresponding to fibrinopeptide B (^1^QGVNDNEEGFFSAR^14^), which is released from the fibrinogen β-chain, and the same peptide with the C-terminal Arg14 missing (^1^QGVNDNEEGFFSA^13^). Fibrinogen is a glycoprotein that helps in the formation of blood clots [24], and consists of two sets of three different chains (α, β, and γ) linked to each other by disulfide bonds. Fibrinopeptide A and B, which are located on the N-terminus of the fibrinogen α- and β-chains, prevent fibrinogen from forming polymers (fibrin). When thrombin cleaves fibrinopeptide A and B from the fibrinogen α- and β-chains, fibrin monomers polymerize to form fibrin. In humans, the normal concentration of fibrinogen is about 1.5–3 g/L. However, increased fibrinogen concentrations have been reported due to heart disease [25], cancer [26], and various types of inflammation [27], among other disorders. Various fragments of fibrinogen have previously been detected in blood, and some have been reported to vary in concentration in different types of disease. For example, the concentration of fibrinopeptide A was found to be altered in the blood of patients with gastric cancer and thyroid cancer [28, 29]. Furthermore, the concentration of a peptide corresponding to residues 605–629 of the fibrinogen α-chain was found to be altered in patients with breast cancer [30].

**Fig 4:**
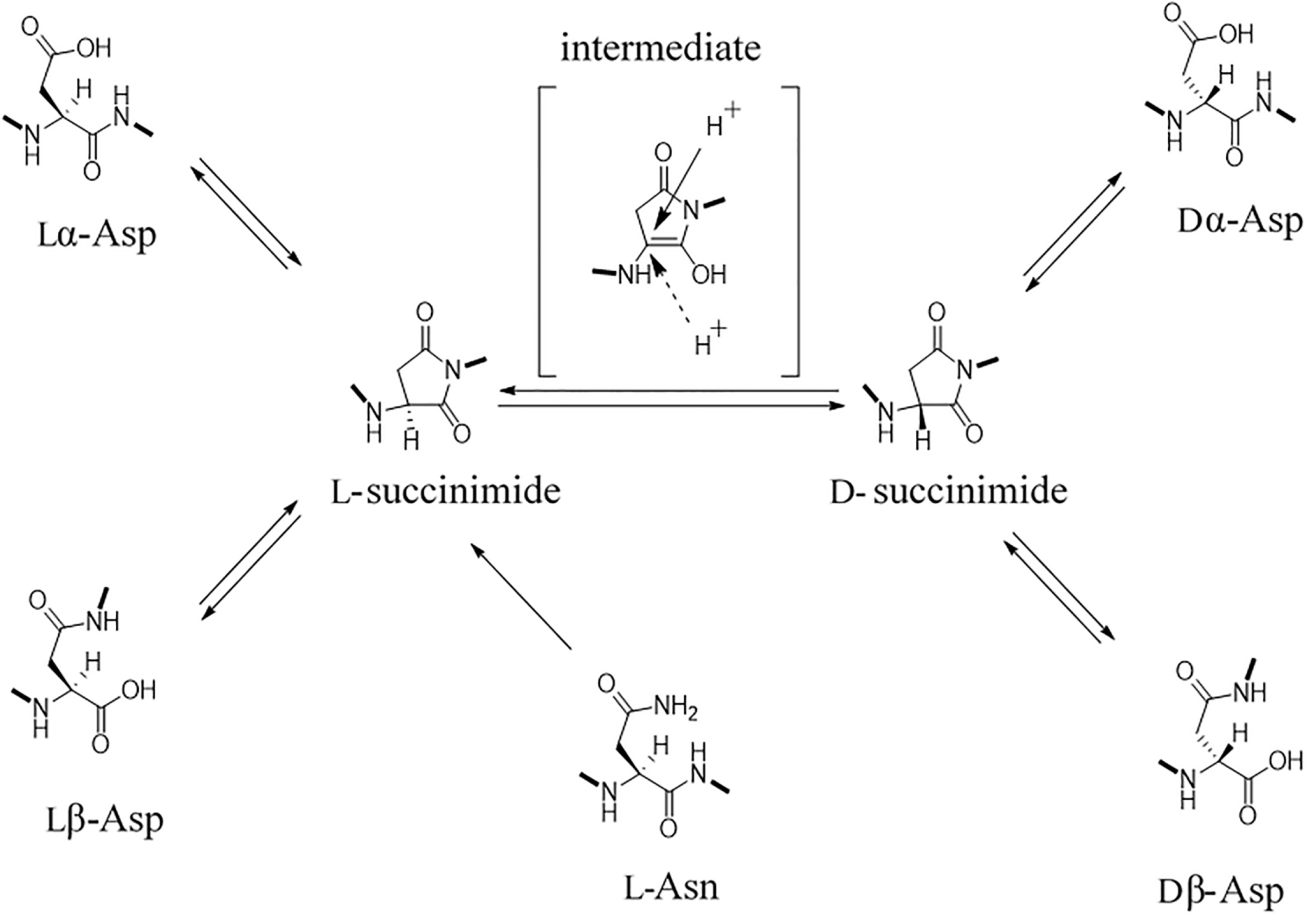
Potential mechanism of isomerization of Asp/Asn in proteins and peptides.

In this study, we identified d-Asp residues in fibrinopeptide B by using diastereomer and LC-MS methods. In general, the presence of d-Asp in living organisms is thought to result from the racemization of Asp residues in proteins. Among all naturally occurring amino acids, the Asp residue is the most susceptible to racemization under physiological conditions. Asp has a carboxyl group in its side chain; therefore, racemization and isomerization of Asp residues in proteins can proceed via a succinimidyl intermediate as follows. (i) If the lone-pair electron of the nitrogen atom of the amino acid following the Asp residue attacks the carboxyl group of the side chain of the lα-Asp residue, then l-succinimide can be generated by intramolecular cyclization. (ii) l-Succinimide can be converted to d-succinimide via an intermediate through keto–enol tautomerism. (iii) d-Succinimide can then be hydrolyzed at either side of its two carbonyl groups to form Dα-Asp or Dβ-Asp; similarly, l-succinimide can be hydrolyzed to form Lα-Asp or Lβ-Asp [16]. Thus, four isomers, lα-Asp, lβ-Asp, dα-Asp and dβ-Asp, can be simultaneously formed in the protein. The rate of succinimide formation is considered to depend on the residue neighboring the Asp residue. Similarly, Asn residues can be deamidated to Asp and isomerized to four isomers, lα-Asp, lβ-Asp, dα-Asp and dβ-Asp (Fig 4).

Fibrinopeptide B contains two Asn residues and one Asp residue, which are joined sequentially as Asn-Asp-Asn. We analyzed the d/l ratio of Asp residues in fibrinopeptide B by the diastereomer method, which involves hydrolysis of the sample as a first step; therefore, Asn residues would be deamidated to Asp residue during this step. As a result, the d/l ratio in fibrinopeptide B, which was calculated as 0.08, was the average for three Asp residues. Thus, if only one Asn or Asp residue in the Asn-Asp-Asn sequence was racemized to d-Asp and the other two Asp residues were not, the actual d/l ratio of that Asn/Asp residue would be three times 0.06 (i.e., 0.18). At present, however, the current methodology is not able to differentiate the d-form content of the individual Asn, Asp and Asp residues in the Asn-Asp-Asn sequence.

It is known that an Asp/Asn residue can be racemized to the d-form when the neighboring amino acid has a small side chain, as found in glycine (Gly), alanine (Ala) or serine (Ser), because succinimide forms easily due to the lack of steric hindrance. Indeed, the amino acid following the remarkable isomeric sites in Asp58 and Asp151 of lens αA-crystallin are Ser59 and Ala152, respectively. However, bulky amino acids have also been observed adjacent to highly isomeric Asp residues, including Asp36–Thr37 and Asp62–Leu63 in lens αB-crystallin [31], Asp4–His5 in lens βB2-crystallin [32], Asp23–His24 of β-amyloid and Asp34–Thr35 in myelin basic protein. These observations indicate that Asp residues may be susceptible to racemization when they are located in flexible regions, as well as when there is low steric hindrance from the adjacent amino acid.

The amino acids adjacent to Asn-Asp-Asn in fibrinopeptide B are, respectively, Asp, Asn and Glu, which are highly sterically hindered. If this region is located in a flexible area of the fibrinogen β-chain, however, then racemization of Asp may occur readily. In other words, racemization may occur in the fibrinogen protein rather than in fibrinopeptide B. At present, it remains unclear whether these residues were isomerized in the protein or isomerized in the truncated peptide, but we cannot exclude the possibility that racemization occurred in the peptide state in blood.

In this study, we successfully detected and identified d-Asp-containing peptides in human blood by two different principle methods: an optical amino acid analysis method and LC-MS. There remains the possibility that other d-amino acid-containing peptides were present in serum; however, it is hard to detect such peptides by the current methodology owing to their low abundance. In addition, because small peptides have high hydrophilicity, it is difficult to separate them by C18 column; therefore, they might not be identified by the methods in this study. Because there are various proteinases and peptidases in the blood, peptides are easily degraded. As a result, the analysis of shorter peptides using a HILIC column might be used to detect other racemized peptides. At present, it is not clear why, among many serum peptides, only d-Asp-containing peptides from the fibrinogen β-chain were detected.

Recent studies have detected post-translational modifications of amino acids in many polypeptides associated with protein aggregation-related disease. The present study has now shown that it is possible to detect racemized peptides in blood. Thus, the analysis of serum samples from patients with protein aggregation-related diseases may facilitate the detection of more abundant racemized peptides. In future studies, proteome research on serum from the standpoint of racemization might enable us to develop new kinds of biomarker.

## Conclusion

We have identified two d-Asp-containing peptides in human serum for the first time. Both peptides derive from fibrinogen B: ^1^QGVNDNEEGFFSAR^14^; and the same peptide with the C-terminal Arg missing, ^1^QGVNDNEEGFFSA^13^. This study will open new avenues in the field of the blood peptidome and fragmentome in proteomics.

## Supporting information

S1 Fig Isolation of peptides from human serum. RP-HPLC chromatograms of serum peptides from the donors aged ~20 and ~40 years. The detailed RP-HPLC conditions are described in Materials and Methods. Arrows indicate Asp-containing peptides.
(TIF)

S2 Fig Determination of the d/l ratio of Asp in synthetic peptide. Elution profile of the enantiomeric separation of Asp derivatives using Boc-l-Cys-OPA. Aspartate residues from the hydrolysates of the synthetic peptide (^1^QGVNDNEEGFFSAR^14^). The d/l ratio of Asp was estimated as 0.02 from the peak areas of the chromatogram.
(TIF)

S3 Fig Determination of the d/l ratio of Asp in human serum-derived peptides. Elution profile of the enantiomeric separation of Asp derivatives using Boc-l-Cys-OPA. Aspartate residues from the hydrolysates of peptide peaks 22 (**a**) and 23 (**b**) in S1(a) Fig (serum sample from the donor aged ~20 years) and peptide peaks 26 (**c**) and 27 (**d**) in S1(b) Fig (serum sample from the donor aged ~40 years) are shown. The d/l ratio of Asp was estimated as 0.08 in all peak by calculating the peak areas of the chromatograms.
(TIF)

S4 Fig Identification of d-Asp-containing peptides by LC-MS/MS analysis. (**a**) LC-MS chromatogram of peak 22 in S1(a) Fig. This peptide was separated into two peaks with the same mass ([M+2H]^2+^ = 777.5), indicating two isomers of the same peptide. (**b**) Tandem mass spectrum of the peak in (**a**). (**c**) LC-MS chromatogram of peak 18 ([M+2H]^2+^ = 698.9) in S1(a) Fig. (**d**) Tandem mass spectrum of the peak in (**c**). (**e**) LC-MS chromatogram of peak 22 in S1(b) Fig. This peptide was separated into two peaks with the same mass ([M+2H]^2+^ = 777.5), indicating two isomers of the same peptide. (**f**) Tandem mass spectrum of the peak in (**e**). (**g**) LC-MS chromatogram of peak 18 ([M+2H]^2+^ = 698.9) in S1(b) Fig. (**h**) Tandem mass spectrum of the peak in (**g**). All peptides were identified as fibrinogen β-chain-specific peptides (peak 22 in S1(a) Fig and peak 26 in S1(b) Fig, ^1^QGVNDNEEGFFSAR^14^; and peak 23 in S1(a) Fig and peak 27 in S1(b) Fig, ^1^QGVNDNEEGFFSA^13^). The N-terminal Gln residue was converted to pyro-Glu in both peptides.
(TIF)
