# Supplementary figures and images for: Identification of ᴅ-amino acid-containing peptides in human serum
Source: PLoS One. 2017 Dec 18;12(12):e0189972. doi: 10.1371/journal.pone.0189972 (PMC5734745; doi:10.1371/journal.pone.0189972)

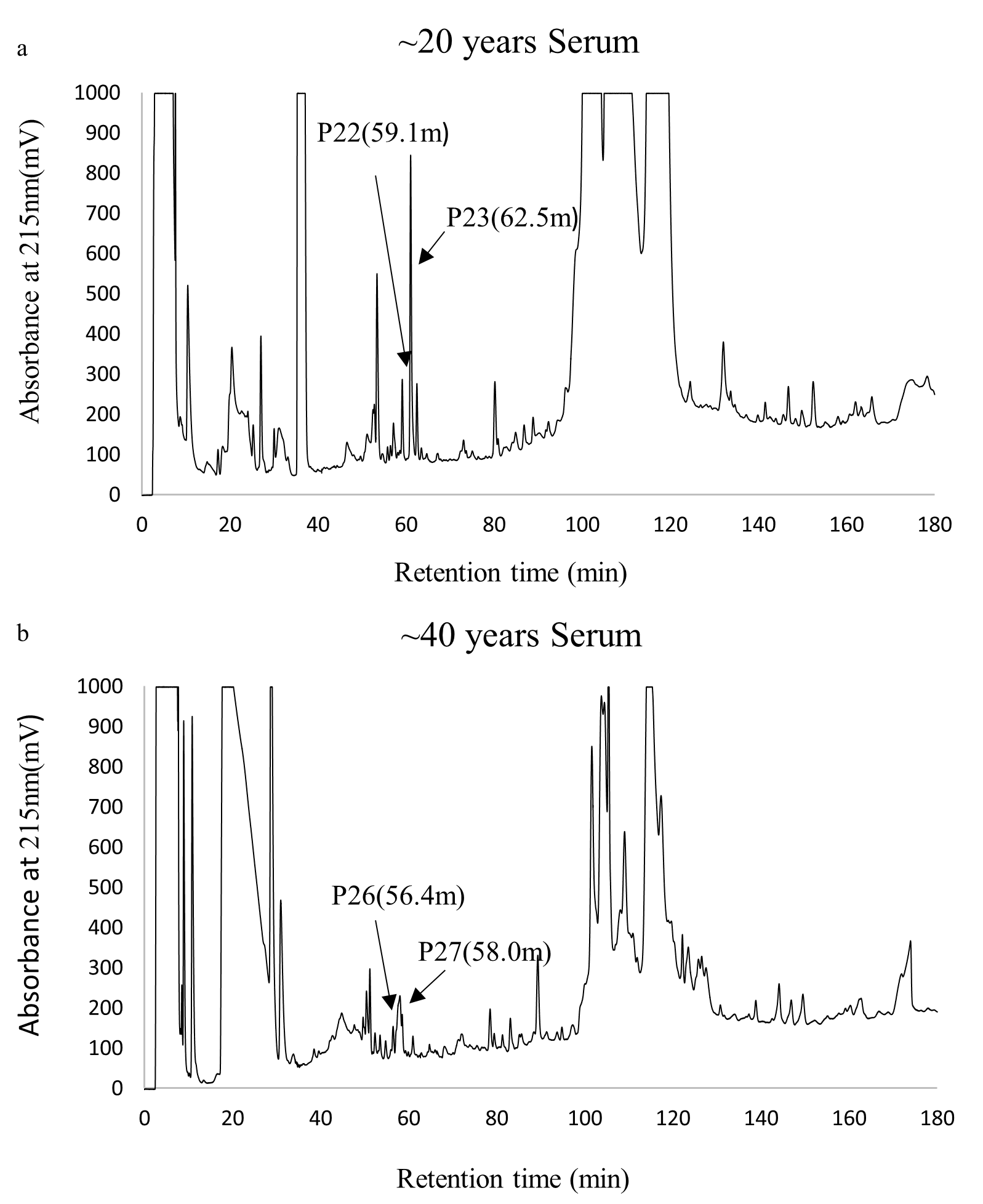

Supplement: S1 Fig — RP-HPLC chromatograms of serum peptides from the donors aged ~20 and ~40 years. The detailed RP-HPLC conditions are described in Materials and Methods. Arrows indicate Asp-containing peptides. (TIF) [file pone.0189972.s001.tif]

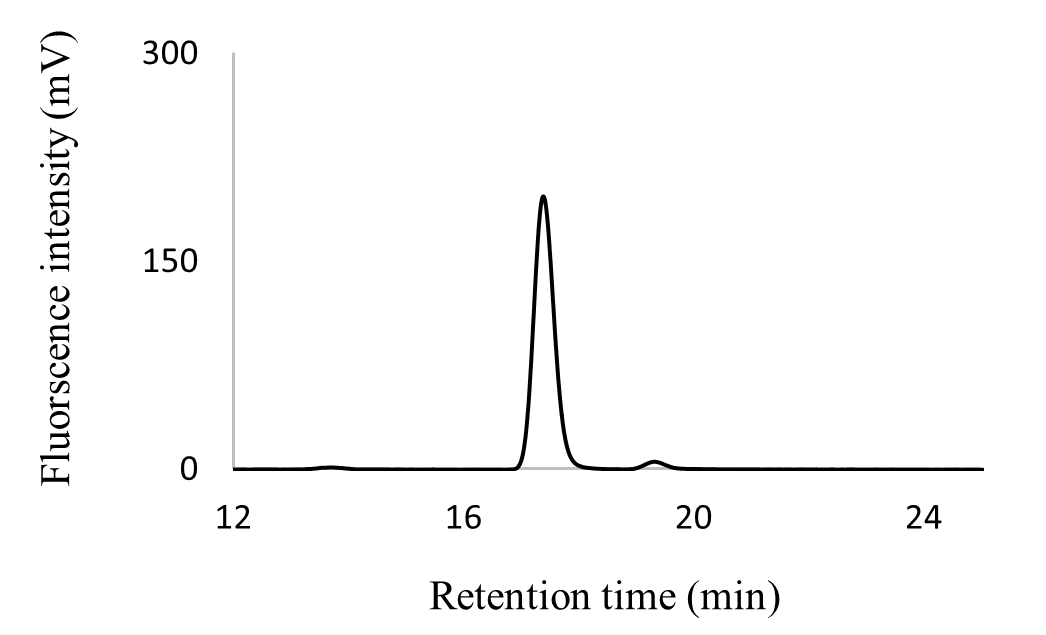

Supplement: S2 Fig — Elution profile of the enantiomeric separation of Asp derivatives using Boc-l-Cys-OPA. Aspartate residues from the hydrolysates of the synthetic peptide (1QGVNDNEEGFFSAR14). The d/l ratio of Asp was estimated as 0.02 from the peak areas of the chromatogram. (TIF) [file pone.0189972.s002.tif]

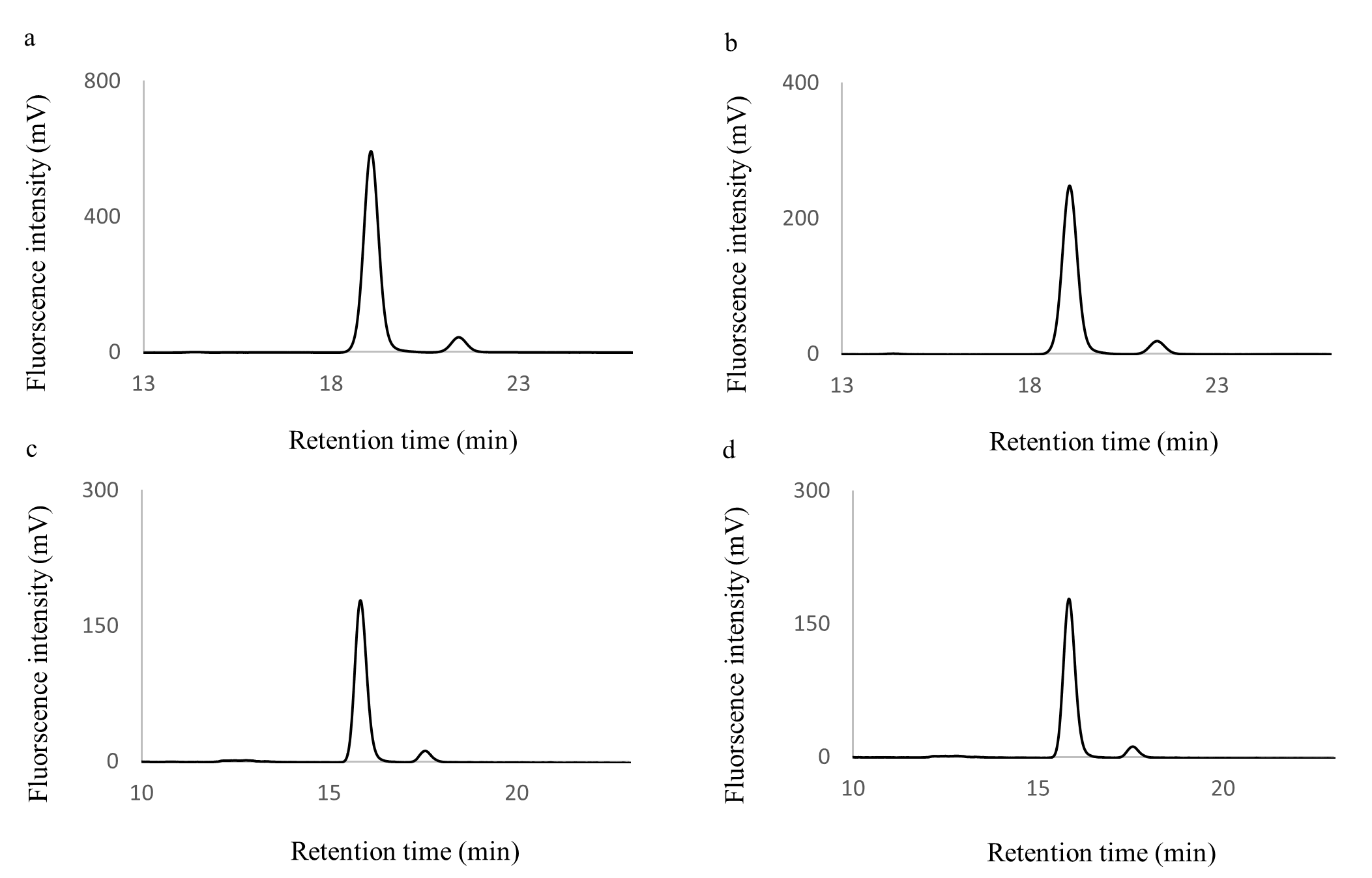

Supplement: S3 Fig — Elution profile of the enantiomeric separation of Asp derivatives using Boc-l-Cys-OPA. Aspartate residues from the hydrolysates of peptide peaks 22 (a) and 23 (b) in S1(a) Fig (serum sample from the donor aged ~20 years) and peptide peaks 26 (c) and 27 (d) in S1(b) Fig (serum sample from the donor aged ~40 years) are shown. The d/l ratio of Asp was estimated as 0.08 in all peak by calculating the peak areas of the chromatograms. (TIF) [file pone.0189972.s003.tif]

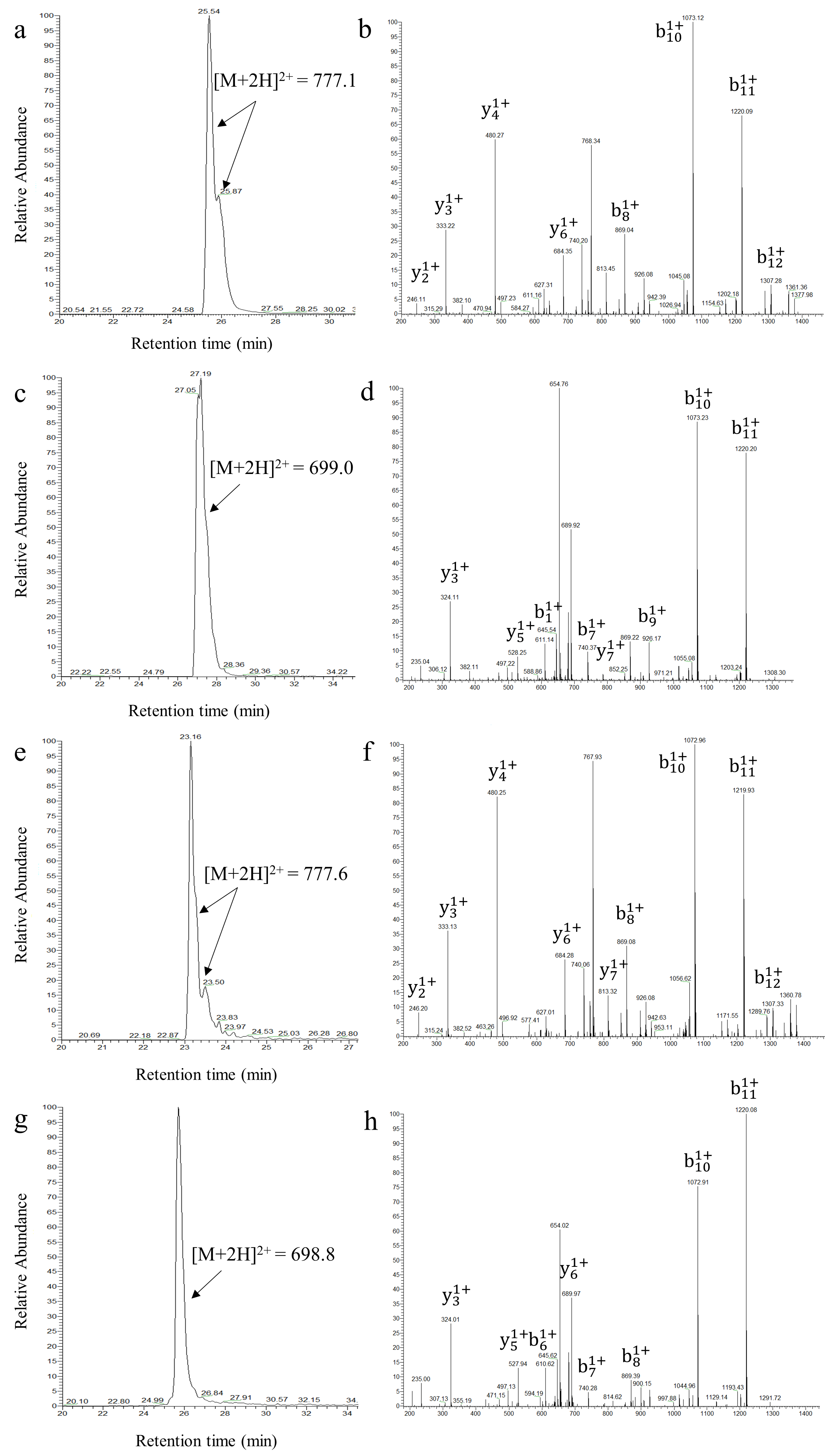

Supplement: S4 Fig — (a) LC-MS chromatogram of peak 22 in S1(a) Fig. This peptide was separated into two peaks with the same mass ([M+2H]2+ = 777.5), indicating two isomers of the same peptide. (b) Tandem mass spectrum of the peak in (a). (c) LC-MS chromatogram of peak 18 ([M+2H]2+ = 698.9) in S1(a) Fig. (d) Tandem mass spectrum of the peak in (c). (e) LC-MS chromatogram of peak 22 in S1(b) Fig. This peptide was separated into two peaks with the same mass ([M+2H]2+ = 777.5), indicating two isomers of the same peptide. (f) Tandem mass spectrum of the peak in (e). (g) LC-MS chromatogram of peak 18 ([M+2H]2+ = 698.9) in S1(b) Fig. (h) Tandem mass spectrum of the peak in (g). All peptides were identified as fibrinogen β-chain-specific peptides (peak 22 in S1(a) Fig and peak 26 in S1(b) Fig, 1QGVNDNEEGFFSAR14; and peak 23 in S1(a) Fig and peak 27 in S1(b) Fig, 1QGVNDNEEGFFSA13). The N-terminal Gln residue was converted to pyro-Glu in both peptides. (TIF) [file pone.0189972.s004.tif]
